# Electrochemical Sensing of Dopamine with a Nafion-Coated Reduced Graphene Oxide/Polypyrrole-Functionalized Magnetic Nanoparticles Composite

**DOI:** 10.3390/mi17080908

**Published:** 2026-07-29

**Authors:** Afef Dhaffouli, Paul E. D. Soto-Rodríguez, Soledad Carinelli, Houcine Barhoumi, José Luis González-Mora, Pedro A. Salazar-Carballo

**Affiliations:** 1 Laboratory of Interfaces and Advanced Materials, Faculty of Sciences of Monastir, University of Monastir, Monastir 5000, Tunisia; dhaffouliafef@gmail.com (A.D.); houcine.barhoumih@yahoo.com (H.B.); 2 Department of Chemistry, Faculty of Sciences of Gafsa, University of Gafsa, Gafsa 2100, Tunisia; 3 Department of Physics, Instituto de Estudios Avanzados (IUDEA), University of la Laguna, 38002 La Laguna, Spain; psotorod@ull.edu.es; 4 Laboratory of Sensors, Biosensors and Advanced Materials, Faculty of Health Sciences, University of La Laguna, Campus de Ofra s/n, 38071 La Laguna, Spain; scarinel@ull.edu.es (S.C.); jlgonzal@ull.edu.es (J.L.G.-M.)

**Keywords:** dopamine, electrochemical sensor, reduced graphene oxide, magnetic nanoparticles, polypyrrole, differential pulse voltammetry

## Abstract

An electrochemical sensor based on a novel composite of electrochemically reduced graphene oxide, polypyrrole-coated magnetic nanoparticles (MNPs@PPy), and Nafion was developed for dopamine (DA) detection. The structural, thermal, and electrochemical properties of the composite were validated through a combination of advanced spectroscopic techniques, thermal profiling, electron microscopy, and impedance analyses. Under optimized conditions, differential pulse voltammetry (DPV) revealed a high sensitivity (1.573 A·M^−1^·cm^−2^, R^2^ = 0.9874) and a limit of detection (LOD) of 5.4 × 10^−9^ M for DA. The sensor displayed excellent selectivity, showing minimal interference from ascorbic acid, uric acid, and acetaminophen. Repeatability and reproducibility were confirmed (coefficient of variation ~8%). Real-sample analysis of urine and blood demonstrated recovery rates between 75 and 116%.

## 1. Introduction

Dopamine (DA) is a neurotransmitter essential for the proper functioning of the central nervous, cardiovascular, and endocrine systems [1]. Dopamine levels are intricately linked with various health conditions including Parkinson’s disease, depression, anxiety, schizophrenia, attention deficit hyperactivity disorder, etc. [2,3,4,5,6]. DA is pivotal in regulating cognitive processes such as sleep patterns, learning, concentration, memory retention, and stress response [7]. Furthermore, DA is integral to the sensation of pleasure and reward, and its release triggers the activation of pleasure centers within the brain, playing a significant role in the development of addictive and suicide behaviors [5,6,8]. New addiction patterns involving DA have emerged related to internet addiction disorders (IAD). Studies have found a significant difference in DA levels between adolescents with IAD and healthy controls, suggesting that DA and its metabolites may play a role in the development of IAD [9]. The dysregulation of DA levels in both adolescents and young adults can lead to increased involvement in issues related to internet addiction, such as depression, anxiety, obsessive-compulsive symptoms, and aggression, which result in adverse outcomes such as school problems and sleep deprivation [10]. Based on the scientific evidence linking DA to numerous biological processes and addictive disorders, there is considerable interest in developing sensitive and selective methods for its detection in clinical specimens such as serum, plasma, urine, and cerebrospinal fluid [11].

Among the different detection methods available, electrochemical detection, including in lab-on-a-chip applications [12], stands out for its simplicity, speed, suitability for point-of-care applications, cost-effectiveness, and high sensitivity [2,13,14,15]. However, some problems arise due to other electroactive interferents, such as ascorbic acid (AA) and uric acid (UA), commonly found in biological matrices. These interferences have oxidation potentials very close to those of DA, which poses a major challenge to the selective detection of DA in biological analyses [16,17,18]. Therefore, it is imperative to carry out rapid and selective determination of DA without interference.

Today, carbon nanomaterials have emerged as prime candidates for constructing electrochemical sensors and biosensors [19]. Various carbon nanomaterials, including carbon quantum dots, single- and multi-walled carbon nanotubes, carbon nanofiber, and graphenic materials, have been extensively investigated. Among these, graphene stands out due to its remarkable potential for sensor applications. This two-dimensional monolayer of graphite has garnered immense interest recently owing to its exceptional properties such as large specific surface area, high electron mobility, robust chemical stability, and impressive electrochemical activity [20]. Graphenic materials have also been widely used as electrode materials in lithium-ion batteries, supercapacitors, fuel cells, (bio)sensors [21,22], and numerous devices and applications. On the other hand, reduced graphene oxide (rGox) is widely used in electrochemical sensors due to its high electrical conductivity and large surface area, which enhance the sensor’s sensitivity and response [23,24,25]. Among various methods, the electrochemical reduction of graphene oxide (Gox) has emerged as an efficient and user-friendly approach for sensor applications [26]. However, graphene’s properties can be further enhanced when it is integrated into more complex structures together with metals [27], metal oxides [28], and conducting polymers [29], increasing its versatility and enhancing its electrochemical properties. In this regard, metal oxides, such as NiO_2_, ZnO, RuO_2_, and SnO_2_, are recognized as active and robust electrocatalysts in sensor and biosensor applications [30,31,32]. However, the widespread adoption of these noble metal oxide catalysts is hampered by their high cost and elemental scarcity. By contrast, Fe_3_O_4_ has emerged as a promising alternative due to its facile, large-scale and low-cost production [33]. Its affordability, magnetic properties, and electrochemical characteristics have attracted growing interest, leading to its widespread use in a variety of areas such as remediation, preconcentration and separation processes [34], and sensing and biosensing applications, among others [35,36]. Magnetic nanomaterials play a crucial role in enhancing sensor platforms, as magnetic nanoparticles offer facile separation, functionalization, and reusability, thereby contributing to accelerated charge-transfer kinetics and an increased electroactive surface area. In this work, the use of polypyrrole-functionalized magnetic nanoparticles leverages these advantages to improve sensor performance. Moreover, conducting polymers, particularly polypyrrole (PPy), polyaniline, polythiophene, and their derivatives, have gained significant attention for their potential in sensor applications due to their high environmental stability, facile synthesis, and excellent electrical conductivity [35,37]. In recent years, hybrid composites combining graphenic materials with other functional nanostructures have shown outstanding electrochemical performance. For instance, a TiO_2_/ruthenium–rGO composite electrode achieved a limit of detection (LOD) of 1.05 nM for ambroxol [38]. Likewise, a GO/g-C_3_N_4_ hybrid-modified electrode detected trace levels of methylparaben and carbamazepine with LODs of 0.82 and 2.82 nM, respectively [39]. These examples highlight the excellent sensitivity achieved by carbon-based hybrid systems, making them highly attractive platforms for the development of advanced electrochemical sensors targeting biologically or medically relevant molecules.

Furthermore, nanocomposites, especially those combining conducting polymers with inorganic metal oxides, metal nanoparticles, carbon nanotubes, or graphene, have recently proven to be effective in biosensing, including electrochemical DA determinations [40,41,42,43]. In this context, Zhang et al. (2014) [40] developed a glassy carbon electrode (GCE) modified with Nafion-coated Fe_3_O_4_@graphene nanospheres (Fe_3_O_4_@GNs), synthesized hydrothermally, for DA detection. The electrode showed excellent electrochemical performance for DA oxidation, with Nafion acting as a permselective barrier to anionic interferents (ascorbic acid (AA) and uric acid (UA)). It exhibited a linear range of 0.020–130.0 μM and a LOD of 0.007 μM [40]. In the same year, Peak-See et al. reported an iron oxide/reduced graphene oxide composite (Fe_3_O_4_/rGOx), synthesized via a one-step in situ wet chemical method, for simultaneous DA and AA detection. The synergistic effect between Fe_3_O_4_ and rGO enhanced the DA signal. The sensor displayed high electrochemical activity and electron transfer efficiency. The differential pulse voltammetry (DPV) showed a linear range of 0.5–100 μM, with a LOD of ca. 0.12 μM [42]. Later, Chen et al. (2019) electrodeposited rGO and overoxidized polypyrrole on a GCE via cyclic voltammetry [41]. The modified GCE facilitated DA and UA oxidation while repelling AA at physiological pH. DA response remained linear with 1.0 mM AA and 0.1 mM UA. Amperometric response to DA extended from 0.4 μM to 517 μM with a LOD of ca. 0.2 μM [41]. More recently, Santhosh Kumar et al. (2021) synthesized iron oxide nanorods coated on PPy/rGOx (Fe_3_O_4_@PPy/rGOx) via chemical reflux [43]. The nanohybrids demonstrated superior electrochemical detection for DA, achieving a LOD of about 0.063 µM and an improved linearity up to 100 µM (R^2^ = 0.994), with excellent DA recoveries (97–98%) in urine sample analyses [43].

In this study, we developed and characterized a composite material consisting of as-prepared graphene oxide sheets, polypyrrole-modified magnetic nanoparticles, and Nafion. The material selection combines rGOx for its high electrical conductivity and large surface area, MNPs@PPy for their fast electron-transfer kinetics and magnetic properties, and Nafion as a permselective membrane to enhance selectivity and minimize interferents. This design aims to provide superior sensitivity, selectivity, and operational stability in dopamine detection. The electrochemical characteristics, as well as the detailed characterization of the composite’s physical properties and chemical composition, were extensively studied and applied to modify GCEs for DA detection. The electrochemical performance and sensing capabilities of the proposed approach were evaluated using DPV for detection applications. Our results demonstrate excellent response toward DA with minimal interference from common substances, enabling detection in the nanomolar range. This is the first report of a ternary composite of rGOx, MNPs@PPy, and Nafion for DA detection, demonstrating nanomolar sensitivity and high selectivity in biological matrices.

## 2. Materials and Methods

### 2.1. Materials and Reagents

Pyrrole monomer (Py), Nafion^TM^ (perfluorinated resin solution, 5% *v*/*v*), graphite, iron (III) chloride (FeCl_3_), iron (II) sulphate heptahydrate (FeSO_4_⋅7H_2_O), iron (III) chloride hexahydrate (FeCl_3_⋅6H_2_O), potassium chloride (KCl), ethanol (EtOH, ≥96%), sodium hydroxide (NaOH), hydrochloric acid (HCl, 37%), sulfuric acid (H_2_SO_4_), potassium permanganate (KMnO_4_), hydrogen peroxide (H_2_O_2_), potassium hexacyanoferrate (II) trihydrate (K_4_[Fe(CN)_6_]·3H_2_O), potassium hexacyanoferrate(III) (K_3_[Fe(CN)_6_]), dopamine hydrochloride, and ascorbic and uric acids were purchased from Sigma-Aldrich (St. Louis, MO, USA). All solutions were prepared with water (resistivity 18.2 MΩ cm). Phosphate buffer (0.1 M) solution was prepared by dissolving monosodium dihydrogen phosphate trihydrate (NaH_2_PO_4_·2H_2_O) and disodium hydrogen phosphate dodecahydrate (Na_2_HPO_4_·12H_2_O) in a 1:1 ratio, both obtained from Merck (Darmstadt, Germany), in water. Phosphate buffer solution (PBS) was used as the supporting electrolyte, with its pH adjusted by adding NaOH or HCl solutions.

### 2.2. Synthesis of Graphene Oxide

Graphene oxide was synthesized using a modified procedure based on previous methods [44,45]. Typically, 0.5 g of graphite powder was dispersed in 30 mL of a (H_2_SO_4_:H_3_PO_4_) mixture (27:3 mL) under moderate stirring. Next, 2.5 g of KMnO_4_ was slowly added, causing a mild exothermic reaction, and the mixture was allowed to react for three days to achieve gentle oxidation of the graphite. The solution’s color changed from dark purplish green to dark brown, indicating the formation of graphite oxide. After three days, 5 mL of 30% H_2_O_2_ was slowly added to stop the oxidation reaction, and the mixture was stirred until gas evolution ceased. The graphite oxide was then purified by centrifugation at 16,000× *g*. The resulting precipitate was washed with 1 M HCl and then five times with deionized water to remove excess manganese salts and acid. During the washing with deionized water, the reaction product exfoliated, as evidenced by the formation of a brown Gox gel. Finally, the gel was dried overnight at 60 °C, yielding laminated GOx as the final material.

### 2.3. Synthesis of Polypyrrole-Modified Magnetic Nanoparticles (MNPs@PPy)

Magnetic nanoparticles were synthesized by a chemical co-precipitation method under a N_2_ atmosphere. The precursor solutions included: (A) 3.3 g of FeCl_3_ and 2.8 g of FeSO_4_·7H_2_O dissolved in 200 mL of 1.2 mM HCl solution, and (B) 300 mL of 1.25 M aqueous NaOH solution. Both solutions were pre-degassed with N_2_. Solution A was added dropwise to solution B at approximately 80 °C under vigorous stirring for 30 min, resulting in the formation of a black precipitate. After an additional 30 min of reaction, the product was magnetically collected and washed with ultrapure water until the supernatant reached a pH of approximately 8.5. Subsequently, the black product was coated with polypyrrole following modified existing methods [46]. The MNPs were dispersed in 200 mL of water at room temperature, and 18 g of FeCl_3_ were added with continuous stirring at 200 rpm for 3 h to accumulate Fe^3+^ ions on the MNPs’ surface via the common ion effect. Then, 40 mL of 5.85 wt% SDS aqueous solution and 1 mL of pyrrole monomer were added, and the mixture was stirred overnight. Finally, the PPy-coated MNPs were magnetically collected, washed with water, a water/ethanol (1:1) mixture, and ethanol (three times each), and dried overnight at 70 °C.

### 2.4. Synthesis of Nafion(Gox/MNPs@PPy)

**Figure 1:**
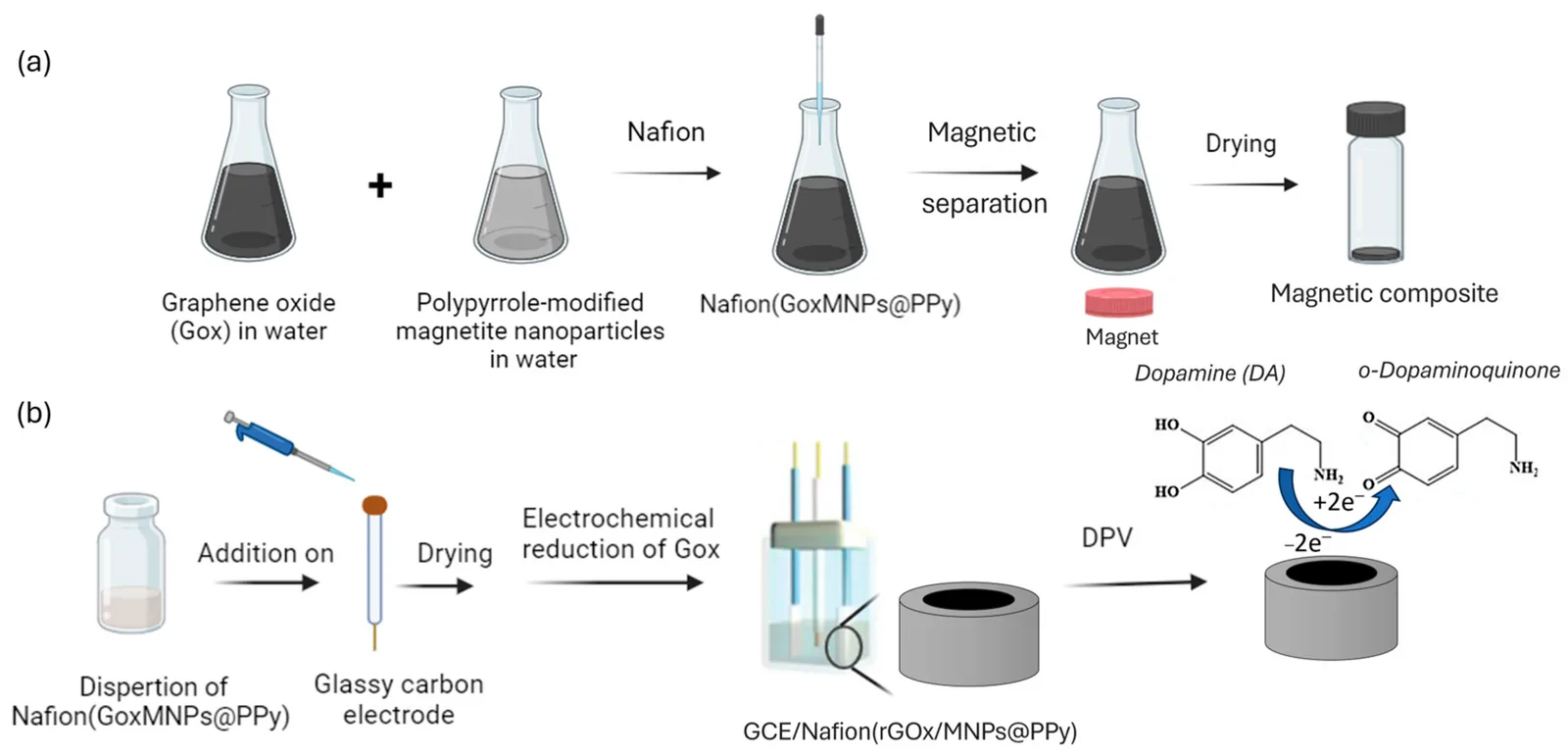
Schematic diagram showing: the synthesis and magnetic separation process of the Nafion(Gox/MNPs@PPy) composite (**a**,**b**) and the electrode assembly protocol, including drop-casting onto a glassy carbon electrode (GCE), drying, electrochemical reduction of Nafion(Gox/MNPs@PPy) to its reduced form Nafion(rGox/MNPs@PPy), and subsequent differential pulse voltammetry (DPV) sensing of dopamine.

The fabrication process began with the preparation of solution (C), in which 100 mg of graphene oxide was dispersed in 20 mL of H_2_O and subjected to sonication for an hour (see Figure 1a). Simultaneously, another solution (D) was created by dispersing 100 mg of PPy-modified MNPS in 20 mL of H_2_O, also undergoing sonication for an hour. The two solutions were then combined to form solution (E), which was stirred for 5 min. Subsequently, 2 mL of Nafion was added to solution (E), and stirring was continued for an additional 1 h to promote composite formation. The resulting composite was magnetically separated and washed twice with H_2_O. Subsequently, the composite underwent a drying process in an oven at 50 °C for 24 h to eliminate any remaining moisture. Finally, the dried composite was meticulously scraped and crushed using a mortar before being stored in Eppendorf tubes for further use.

### 2.5. Preparation of the GCE/Nafion(rGox/MNPs@PPy) Electrode

First, the Nafion(Gox/MNPs@PPy) composite was dispersed in water (1 mg/mL) and sonicated for 10 min (see Figure 1b). Before surface coating, the glassy carbon electrodes (3 mm diameter) were sequentially polished with 1.0 µm, 0.3 µm, and 0.05 µm alumina powder, then rinsed with deionized water, ethanol, and deionized water for 3 min each. This polishing and cleaning process is essential to remove surface impurities and ensure a smooth, uniform electrode surface, which enhances the reproducibility and sensitivity of electrochemical measurements. Then, 5 µL of the prepared solution was deposited on the surface of the GCE and the electrode was dried at room temperature. The graphenic material was then reduced by cyclic voltammetry (CV) scanning in PBS pH 5.0, in a potential range from 0.0 to −1.7 V versus Ag/AgCl, giving the reduced form of the composite, Nafion(rGox/MNPs@PPy).

### 2.6. Characterization of As-Prepared Samples and Electrochemical Measurements

The examination of sample morphology and microstructure was conducted through Transmission Electron Microscopy (TEM) and obtained using a JEOL 2100 microscope at 200 kV (JEOL Ltd., Tokyo, Japan). For TEM analysis, the synthesized material was dispersed in absolute ethanol using an ultrasonic bath for 15 min, and a drop of the stable suspension was deposited onto a carbon-coated grid and allowed to dry completely at room temperature before imaging. The surface morphology and elemental composition were analyzed by Scanning Electron Microscopy (SEM) and a ZEISS EVO 15 microscope (Carl Zeiss Microscopy GmbH, Oberkochen, Germany). The system was equipped with an Oxford X-MAX 50 mm^2^ energy-dispersive X-ray spectroscopy (EDS) microanalyzer (Oxford Instruments, Abingdon, UK) for elemental determination and mapping. For sample preparation, the composite was dispersed in ethanol, and a single droplet was drop-casted onto a copper substrate. The sample was allowed to dry overnight under ambient conditions prior to its introduction into the SEM vacuum chamber. X-ray Diffraction (XRD) patterns for the powder samples were acquired utilizing a Philips Panalytical X’Pert powder diffractometer Malvern Panalytical, Almelo, Netherlands) with CuKα radiation (λ = 1.540 Å) in the 2θ range spanning from 5° to 80° with a scan step size of 0.05° and counting time of 1 s per step. Raman analysis was carried out using a Renishaw InVia micro-Raman (μRaman) system (Renishaw plc, Wotton-under-Edge, UK) with a 532 nm He-Ne laser operating at a low laser power (<1 mW) to prevent sample degradation, with the dried powder samples placed directly onto clean glass slides. Magnetic measurements were performed using a Quantum Design MPMS-WL-5 superconducting quantum interference device (SQUID) magnetometer (Quantum Design Inc., San Diego, CA, USA) at 300 K by sealing approximately 10 mg of dry powder inside a non-magnetic gelatin capsule. Fourier-transform infrared (FT-IR) spectroscopy was performed using a Jasco FT/IR-6800 spectrometer (JASCO International Co., Ltd., Tokyo, Japan) equipped with an Attenuated Total Reflection (ATR) accessory. Dry powder sample was placed directly onto the surface of the diamond crystal and firmly clamped. Spectra were collected in the wavenumber range of 4000–400 cm^−1^, using the ambient air spectrum as the background.

Electrochemical measurements were conducted using a computer-controlled Autolab PG potentiostat/galvanostat (AUT 83965) using a conventional three-electrode cell configuration, with the working electrode being a modified GCE, a platinum wire serving as the counter electrode and an Ag/AgCl electrode (saturated with KCl) as the reference electrode. Electrochemical impedance spectroscopy (EIS) analyses were carried out using a solution with 5 mM Fe(CN)_6_^3−/4−^ redox probe and containing 0.1 M KCl (applied potential = +0.2 V vs. Ag/AgCl; frequency range = 0.1–10^5^ Hz, signal amplitude of the sinusoidal wave = 10 mV).

The DPV analysis involved two sequential phases: (1) the polarization of the working electrode (GCE/Nafion(rGox/MNPs@PPy)) at a potential of −0.4 V vs. Ag/AgCl for 5 min to preconcentrate cationic DA on the sensor surface, and (2) the application of a potential ramp in the range from −0.4 V to +0.8 V (pulse amplitude = 50 mV, pulse width = 50 ms, and scan rate = 20 mV/s) to oxidize the accumulated DA into dopamine-o-quinone.

## 3. Results

### 3.1. Material Characterization

The chemical, structural, and morphological properties of the Nafion(Gox/MNPs@PPy) composite were characterized through Fourier transform infrared spectroscopy (FT-IR), XRD, Raman spectroscopy, thermogravimetric analysis (TG), EIS, and TEM, confirming the successful synthesis of the composite.

**Figure 2:**
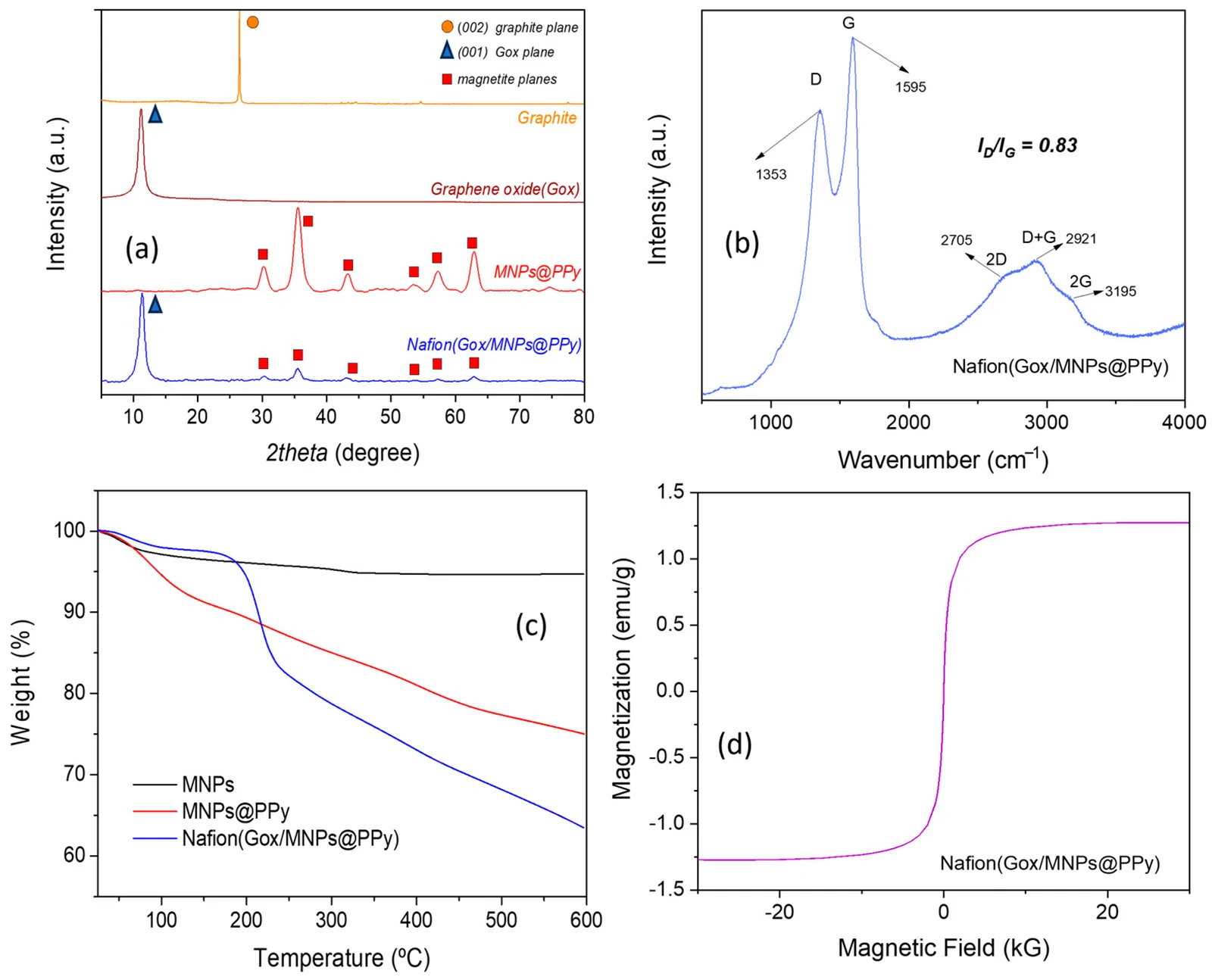
Characterization of precursors and the Nafion(Gox/MNPs@PPy) composite: (**a**) XRD patterns, (**b**) Raman spectrum, (**c**) thermogravimetric analysis (TGA) curves, and (**d**) magnetization curves measured by SQUID (Quantum Design Inc., San Diego, CA, USA).

Figure 2a shows the diffractogram for the proposed composite, its precursors, and intermediate products. The diffractogram for graphite flakes showed a prominent and sharp peak at 26.5°, corresponding to the (002) planes with an inter-layer spacing of 0.336 nm. Following the chemical oxidation of the pristine graphite, the diffraction peak of the graphene oxide shifted to a lower angle, around 11.2°, which corresponds to the (001) planes and an inter-layer spacing of ca. 1.01 nm [27]. This increase in inter-layer spacing can be explained by the intercalation of water molecules and the formation of oxide functional groups (such as epoxides, hydroxyls, and carboxyls) on the carbon basal plane during the chemical oxidation process. The MNPs@PPy presented diffraction peaks at 29.98°, 36.41°, 42.95°, 53.23°, 57.03° and 62.60°, corresponding to the (220), (311), (400), (422), (511) and (440) Bragg reflections, respectively [35]. This pattern confirmed the polycrystalline nature of Fe_3_O_4_ with a cubic spinel structure. The XRD pattern, observed for the Nafion(Gox/MNPs@PPy) composite, confirmed the presence of both precursors in the final product (MNPs and Gox), suggesting that the crystalline structure of the MNPs and the chemical composition of the Gox sheets were not affected during the mixing process.

The Raman spectrum of the composite is depicted in Figure 2b. It shows the typical vibrational characteristics of graphene oxide reported in the literature [47]. Two main peaks are observed: one at 1353 cm^−1^, corresponding to the D mode, which is associated with structural defects and disorder in the carbon lattice, and another at 1595 cm^−1^, corresponding to the G mode, related to the vibration of carbon atoms in sp^2^ bonds within the hexagonal plane. The relative intensity of the D and G modes (I_D_/I_G_) was 0.83, suggesting that the graphene underwent a mild modification process, preserving its sp^2^-ordered structure and limiting defect introduction. This mild synthesis enhances key properties, such as high electrical conductivity, greater chemical stability, and reduced electrochemical noise, ensuring consistent performance and making it ideal for advanced sensor applications. Additionally, the spectrum exhibits contributions from overlapping lower-intensity bands: the 2D band at 2705 cm^−1^, the 2G band at 3195 cm^−1^, and a shoulder between these peaks at 2921 cm^−1^. These features are associated with structural and chemical defects in graphene oxide. These results align with those reported in the literature for graphene oxide, confirming the structure of the analyzed material [48].

Thermal analysis was performed to confirm and estimate the relative composition of materials in the composite, and the results are shown in Figure 2c. The weight loss of unmodified MNPs was very low, and after a temperature of about 200 °C, a weight loss of about 3% of the initial weight was observed and could be ascribed to the removal of physically adsorbed water. However, the MNPs@PPy curve shows the first weight loss stage below 100 °C (associated with the weight loss of adsorbed water); after that, an almost constant weight loss is observed and attributed to the thermal decomposition of the organic layer (PPy shell) [49].

Nafion(Gox/MNPs@PPy) composite showed a more complex thermogram. As with the previous ones, the weight loss below 100 °C might be ascribed to evaporation of adsorbed water. The next temperature range, between 200 and 300 °C, presented a weight loss of ca. 17% and was ascribed to trapped water in the polymeric materials (Nafion and PPy) and in the graphene oxide interlayers. Finally, a continuous weight loss is observed due to the thermal decomposition of the organic materials (graphene sheets and polymeric materials) until they reach a value of ca. 60%.

Figure 2d shows the Magnetization (M) vs. Magnetic Field (H) curve (M-H curve) for the Nafion(Gox/MNPs@PPy) composite. The absence of a hysteresis loop confirms the superparamagnetic behavior of the composite material [50]. This behavior is attributed to the small size of the MNPs@PPy, which is sufficiently small to form magnetic domains that behave as a single magnetic domain (monodomain). The strategic incorporation of these magnetic nanoparticles provides crucial advantages to the electrochemical dopamine sensing platform. Thanks to their superparamagnetic nature, the composite exhibits a strong magnetic response under an external magnetic field but retains zero remanence when the field is removed. Furthermore, the integration of MNPs within the PPy matrix provides a high surface-to-volume ratio and acts as a synergistic electrochemical modifier. This synergetic effect increases the electroactive surface area and facilitates faster electron transfer kinetics for dopamine oxidation, directly boosting the sensor’s sensitivity and lower detection limits. This characteristic enables the Nafion(Gox/MNPs@PPy) composite to be easily controlled with a magnet during subsequent processes, and opens a plethora of possibilities for integration with other functional materials and facilitating its incorporation into various technological applications such as drug delivery [51], bio-magnetic imaging [52], environmental applications [53], or the fabrication of smart electronic devices [54]. Furthermore, the integration of MNPs within the polymeric and graphene-based matrix provides an exceptionally high surface-to-volume ratio. Crucially, the intercalation of these nanoparticles prevents the irreversible restacking and collapse of the graphene sheets, preserving their outstanding native properties. This structural stabilization, combined with the synergistic effect of the components, significantly increases the electroactive surface area and facilitates faster electron transfer kinetics for dopamine oxidation. Consequently, these features directly boost the sensor’s sensitivity and lower its detection limits [55].

**Figure 3:**
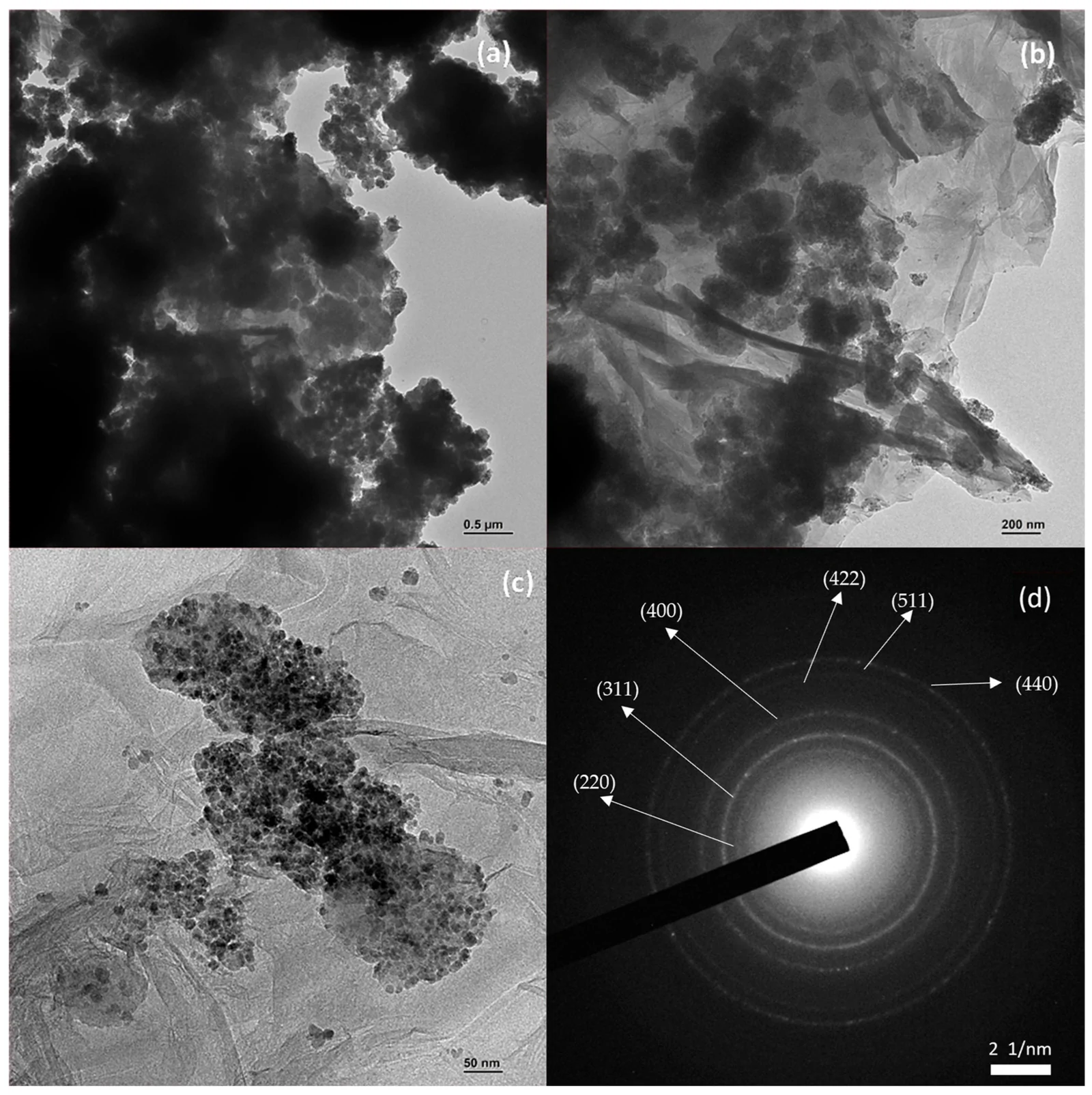
Transmission electron microscopy (TEM) characterization of the Nafion(Gox/MNPs@PPy) composite: (**a**–**c**) bright-field TEM micrographs at increasing magnifications, and (**d**) selected area electron diffraction (SAED) pattern with indexed Miller indices.

The TEM image, Figure 3a, reveals polypyrrole-modified MNPs with an almost spherical configuration and a polydisperse nature. Moreover, the MNPs@PPy were well distributed over the large surfaces of the Gox sheets. A detailed observation, Figure 3b, clearly confirms the incorporation of the MNPs in the PPy matrix, with a core–shell nanostructure within which numerous nanoparticles are embedded in the polymeric matrix. Figure 3c shows a high-magnification TEM image where small MNPs are well observed with an average size of about 10 nm. The last value is in good agreement with previous work of our group using a similar synthetic route [46,56]. Moreover, the use of a polymeric matrix did not change either the spherical shape or size of the MNPs. Finally, Figure 3d depicts the Selected Area Electron Diffraction (SAED) pattern of the Nafion(Gox/MNPs@PPy) composite, where the concentric rings observed display the diffraction pattern typical of an isotropic polycrystalline material. In particular, these diffraction rings can be indexed to the (220), (311), (400), (422), (511), and (440) crystallographic planes, which correspond to the characteristic lattice reflections of the MNPs’ inverse spinel structure. This assignment shows an excellent correlation with the diffraction peaks previously observed in the XRD pattern (Figure 2a), confirming the highly crystalline nature and consistency of the crystal phases across both characterization techniques.

**Figure 4:**
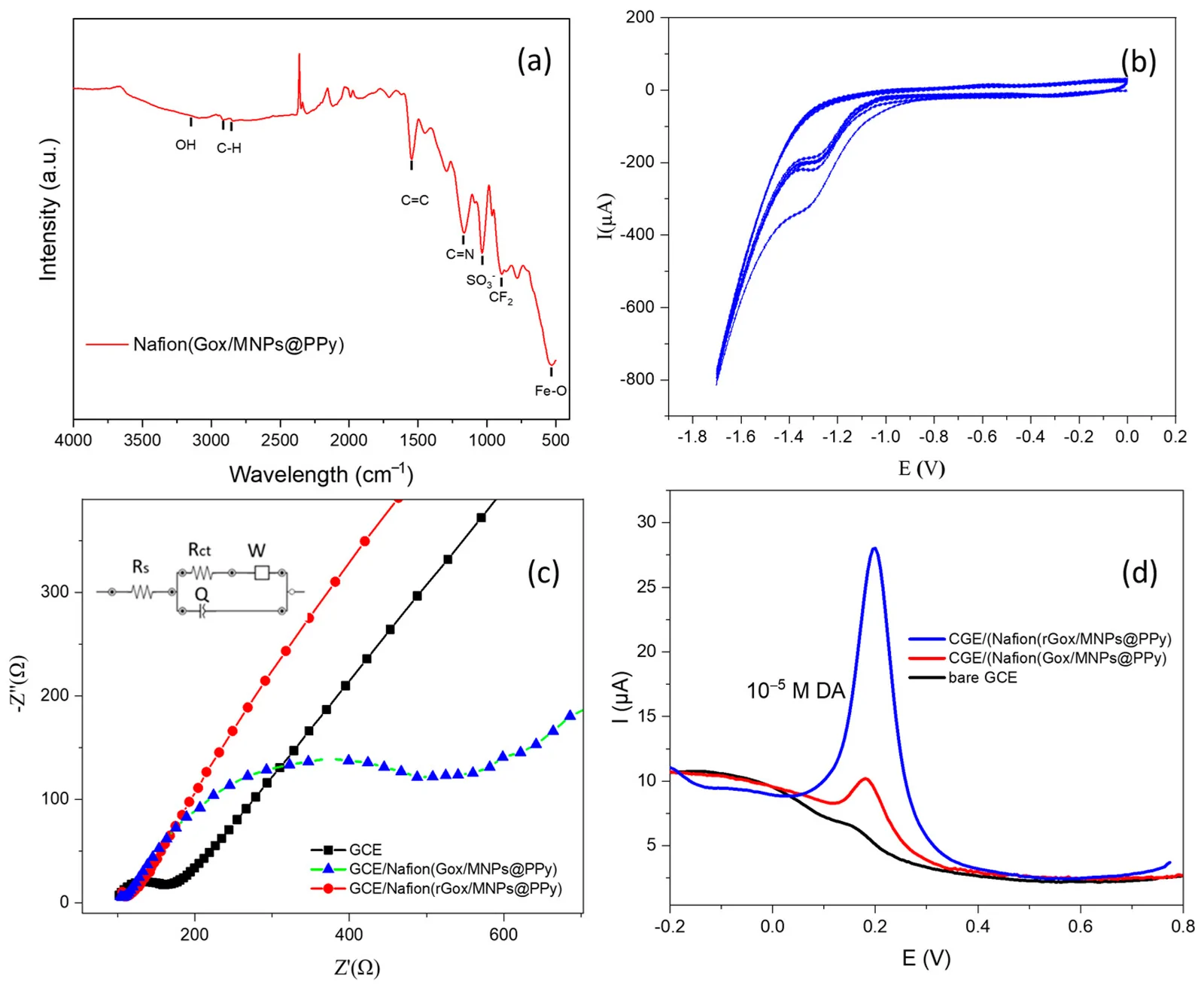
(**a**) FT-IR spectrum of Nafion(Gox/MNPs@PPy) composite; (**b**) electrochemical reduction of Nafion(Gox/MNPs@PPy) composite to its reduced form (Nafion(rGox/MNPs@PPy)) onto the GCE surface; (**c**) EIS spectra of bare GCE, GCE/Nafion(Gox/MNPs@PPy) and GCE/Nafion(rGox/MNPs@PPy) using a 5 mM Fe(CN)_6_^3−/4−^ redox probe (implemented with 0.1 M KCl); (**d**) DPV for dopamine of bare GCE, GCE/Nafion(Gox/MNPs@PPy) and GCE/Nafion(rGox/MNPs@PPy). Data were recorded using a 7 µL casting solution, a 2 h drying time, and a 3 min accumulation time in a PBS pH 7.0 buffer.

Finally, Figure 4a presents the FT-IR spectrum of the Gox-MNPs-PPy-Nafion composite, acquired using Attenuated Total Reflectance in the spectral range of 4000–500 cm^−1^. The spectrum reveals the characteristic vibrational modes associated with the functional groups of graphene, magnetite (Fe_3_O_4_), polypyrrole, and Nafion, confirming the successful incorporation of each component into the hybrid material. A broad absorption band around ~3400 cm^−1^ is observed, which can be attributed to O–H stretching vibrations, likely arising from surface hydroxyl groups on graphene oxide or residual moisture. In addition, other peaks related to C-H groups are identified in this region. Between 1600 and 1450 cm^−1,^ several absorption peaks emerged corresponding to C=C and C–N stretching vibrations, which are characteristic of the conjugated structure of polypyrrole. Distinctive bands between 1250 and 1050 cm^−1^ are associated with the symmetric and asymmetric stretching modes of the sulfonate (SO_3_^−^) groups in Nafion, along with CF_2_ stretching vibrations, indicative of the polymer backbone. Finally, a prominent absorption band around ~580 cm^−1^ was ascribed to the Fe–O stretching vibration and confirmed the presence of magnetite nanoparticles in the composite.

#### Electrochemical Conditioning of the Working Electrode

Before use, the composite-modified GCEs were properly conditioned to optimize their conducting and electrochemical response [24,25]. To this end, each GCE/Nafion(Gox/MNPs@PPy) electrode was cycled (n = 6) in a potential range from 0 to −1.7 V vs. Ag/AgCl at 100 mV/s to obtain the reduced form of the composite and the GCE/Nafion(rGox/MNPs@PPy) configuration. Figure 4b shows the CVs during this step, where a prominent cathodic peak appears at −1.4 V vs. Ag/AgCl, corresponding to the electrochemical reduction of the oxygen functional groups in Gox. In the subsequent cycles, the cathodic peak became much smaller, and it was stabilized, indicating that the Gox has been completely converted to the reduced form (rGox). The absence of the corresponding anodic peak in the CV curves shows that the electrochemical reduction of Gox is irreversible and that rGOx cannot be oxidized in the experimental potential range [57,58].

Electrochemical impedance spectroscopy analysis was used to confirm the electrochemical reduction of the composite and its characteristics. Figure 4c shows the EIS spectra for the oxidized and reduced form of the composite (data for bare GCE is shown for comparison purposes). All EIS curves were fitted using the equivalent circuit shown in Figure 4c *(inset)*; for details, see [59], and the charge-transfer resistance (semicircle diameter, Rct) was obtained for the different configurations. The freshly polished electrode exhibited a resistance (Rct value) of about 84 Ω, demonstrating the excellent electrochemical properties of the GCE for analytical applications. After modification with the Gox-based composite, the electrochemical resistance increased to approximately 539 Ω, indicating a significant rise in resistance due to the presence of the Nafion(Gox/MNPs@PPy) composite on the electrode surface, highlighting the insulating properties of its oxidized form of graphene. Following electrochemical conditioning via electrochemical reduction, the reduced composite (Nafion(rGox/MNPs@PPy)) improved the electrode’s electrochemical performance; thus, the characteristic semicircular feature in the impedance spectrum, typically associated with resistive processes, disappeared, leaving only the diffusional component (straight-line portion). This change highlights the enhanced electron transfer and improved conductivity achieved after the reduction of the composite, presumably owing to the elimination of oxygen groups in the graphene defects and the restoration of a graphitic network of sp^2^ bonds [60].

To confirm the analytical advantage of the composite, we studied the response against DA at different stages during the assembly process. DPV was used for this purpose due to its superior current sensitivity, enhanced peak resolution, and lower detection limit in comparison to the cyclic voltammetry method, owing to the effective elimination of background capacitive current [61]. Figure 4d shows the DPV response of bare GCE, GCE/Nafion(Gox/MNPs@PPy), and GCE/Nafion(rGox/MNPs@PPy) electrodes against DA. The GCE/Nafion(rGox/MNPs@PPy) response shown in this figure is the response after optimizing the main experimental parameters (*see above*). DPV reveals the oxidation peak of DA at approximately +0.2 V vs. Ag/AgCl, with a notably weaker response for the bare GCE compared to the composite-modified electrodes. Initially, the composite in its oxidized form exhibits a response superior to that of the bare electrode. However, after applying the electrochemical reduction step, a significant enhancement in the sensor’s performance is observed. This enhancement in sensitivity can be attributed to the improved electrochemical properties of the reduced composite, restoring the graphitic structure of graphene by eliminating oxygen-containing functional groups, facilitating faster electron exchange at the electrode surface, and improving DA oxidation.

**Figure 5:**
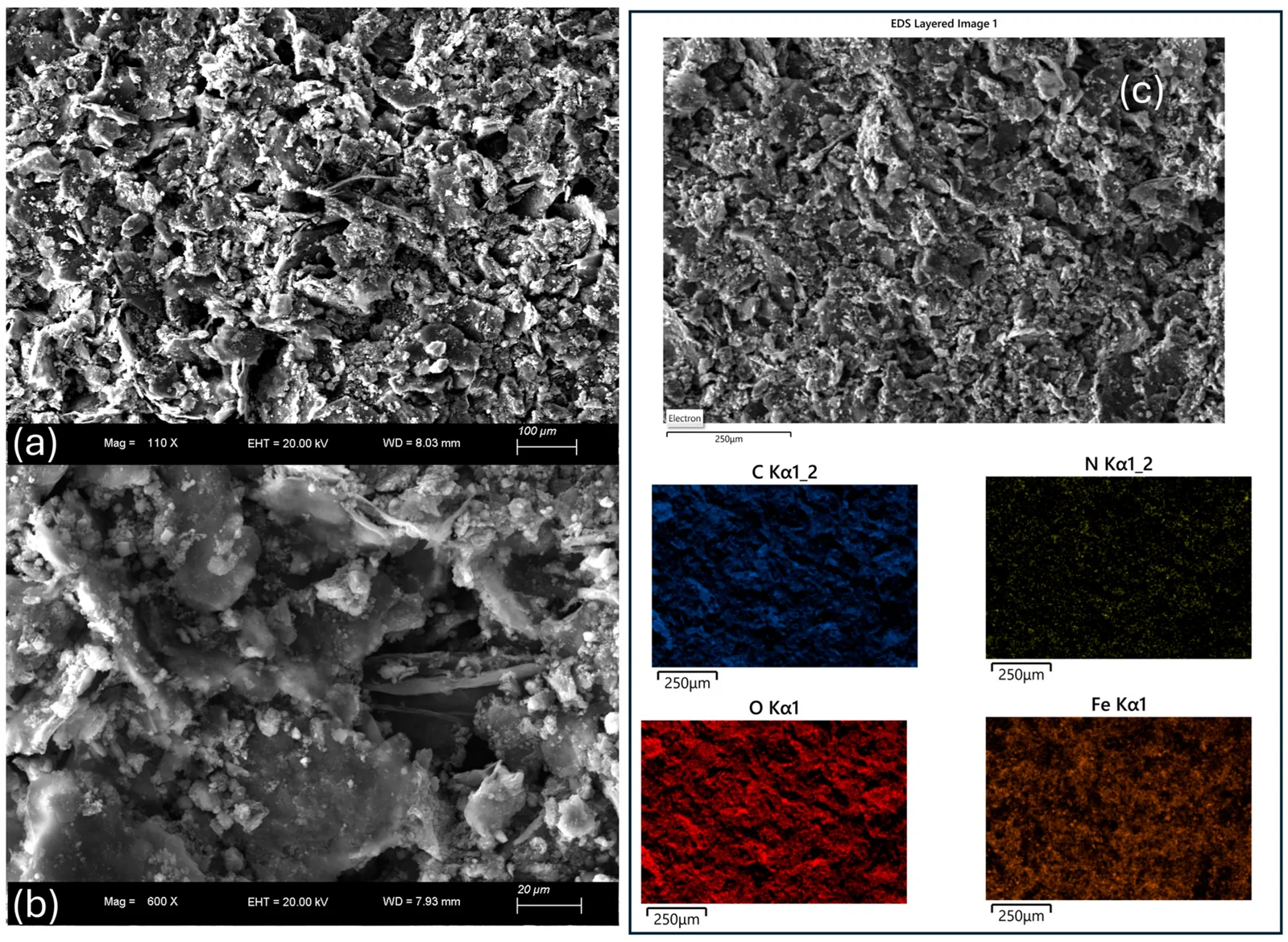
SEM micrographs at low (**a**) and high magnification (**b**), and EDS elemental mapping (**c**) of the Nafion(Gox/MNPs@PPy) composite film for Carbon (C), Nitrogen (N), Oxygen (O), and Iron (Fe) elements.

To evaluate the morphological features, uniformity, and compactness of the composite film, SEM and EDS mapping analyses were performed. As shown in Figure 5a, the low-magnification SEM micrograph demonstrates a highly uniform, continuous, and homogeneous coverage across the surface without macroscopic fractures or exposed substrate areas. At higher magnification (Figure 5b), the microstructure reveals a dense, tightly packed, and compact granular morphology characteristic of the composite matrix. To confirm the homogeneous spatial distribution of the components within this compact matrix, elemental mapping was carried out over the region in Figure 5c. The elemental maps and composition analysis (Supplementary Table S1) reveal a clear and overlapping distribution of Carbon (C (56.67 wt%), blue), Nitrogen (N (2.62 wt%), yellow), and Oxygen (O (26.52 wt%), red), which constitute the foundational organic and polymeric framework of Nafion^TM^, PPy, and Gox. Crucially, the Iron map (Fe (12.42 wt%), orange) displays a highly uniform and well-dispersed distribution across the entire analyzed area without significant agglomeration of MNPs, ensuring uniform distribution and structural properties. Crucially, Copper (Cu) is detected at a remarkably residual level of only 0.14 wt%, which stems from the underlying SEM slide substrate. The lack of interconnected porosity or visible micro-defects at this scale, and the combination of the Nafion^TM^ polymeric film, slows down diffusion pathways of anionic species, ensuring excellent performance against common biological electrochemical interferents.

### 3.2. Optimization of the Main Experimental Parameters for Sensing Applications

#### 3.2.1. Electrochemical Reduction of the Graphemic Composite

To improve the electrochemical response of the Nafion(rGox/MNPs@PPy) composite, the electroreduction process was studied in detail. To this end, the pH of the working solution and the number of reduction cycles were optimized because they can modify the extent of the reduction process (from Gox to rGox). The reduction step was performed with CV at a scan rate of 50 mV/s. After completing the reduction process, the electrode was rinsed with distilled water and dried at room temperature for one hour to prevent the loss of the deposited compound. The response of the sensor to DA was then recorded with DPV.

##### Effect of the Number of Cycles During the Electrochemical Reduction

Firstly, the number of cycles (*n*) during the electrochemical reduction step was examined, and the pH value was fixed to a pH value of 5.0. Supplementary Figure S1 (see Supplementary Materials) shows a clear increase in the DA detection by DPV until *n* = 6, then the sensor response decreases, stabilizing at a higher number of cycles. Such behavior can be ascribed to the better conductivity and electrochemical activity of the rGox; however, the overreduction of the composite can affect the sensor response and the DA sensitivity. The loss of sensitivity may arise from structural and chemical changes in the material such as the removal of oxygen functional groups crucial for DA adsorption, change in the hydrophobicity of the composite, the appearance of structural defects, and/or surface passivation, compromising electron transfer and sensitivity [26]. For the subsequent analysis, six cycles were used during the electrochemical reduction step.

##### Effect of the pH of the Solution During the Electrochemical Reduction

Supplementary Figure S2 shows the sensor response against DA for different composites reduced at varying pH values. Data confirm the better analytical performance of the composite when electrochemical reduction occurs in the pH range 3.0–5.0. This can be justified by the electrochemical reduction of carboxyl, aldehyde, and other oxygen-containing groups, which involves their protonation [26,62]. At higher pH, the absence of protons may limit the reduction of the composite, while at a very low pH the overreduction of the composite may have a negative effect on the electroactivity against DA, as shown in the previous section. For the subsequent analysis, a pH of 3.0 was used during the electrochemical reduction process.

#### 3.2.2. Optimization of the Electrochemical Detection of DA

To optimize the sensor’s performance, the main experimental variables were fine-tuned. These included the amount of deposited material, the pH of the working solution, the accumulation period during DPV, and the drying/curing time of the electrode during its preparation. The results of these optimizations are summarized in Table 1 and shown in the Supplementary Materials (Figures S3–S6). For details on similar optimizations, refer to the following reference [63].

The effect of the deposited amount of the composite on the sensor surface was investigated by varying volumes of Nafion(rGox/MNPs@PPy) dispersion (1 mg/mL). The electrochemical response increased with the deposited volume from 3 to 7 μL; after that, the sensor response decreased due to increased electron transfer resistance from an excessive amount of the material deposited on the sensor’s surface. The drying time of the sensor coating also may affect the oxidation current response due to several factors such as irregular coatings, reduction of the active surface (aggregation or agglomeration effects), and increase of the impedance due to loss of moisture, etc. The analytical signal increased between 1 and 2 h of drying but decreased for longer durations, indicating that optimal drying, which ensures a uniform and stable coating, occurs at 2 h.

The pH of the working solution significantly influences the electrochemical behavior of dopamine, particularly because its redox reactions involve proton-coupled electron transfer processes [64]. It is also well established that DA is chemically unstable under neutral to basic conditions, where it undergoes oxidation and polymerization reactions that degrade the signal quality and reproducibility [65]. To evaluate the optimal operating conditions, we studied the electrochemical response of the GCE/Nafion(rGox/MNPs@PPy) sensor in 0.1 M PBS over a pH range of 3.0–9.0. The results showed that the sensor exhibited the highest and most stable peak current at pH 6.0–7.0, as confirmed in Supplementary Figure S6a. This pH not only ensures maximum electrochemical response but also reflects physiological conditions relevant for clinical applications. At acidic pH, increased protonation of DA and the sensor surface reduces signal stability and selectivity. In contrast, at higher pH values, DA degradation accelerates, leading to lower sensitivity due to polymerization and formation of electro-inactive by-products. Therefore, PBS at pH 7 was selected as the optimal supporting electrolyte for its balance of electrochemical performance, DA stability, and biological relevance. Moreover, the peak potential shifts linearly toward less positive values as the pH increased from 4.0 to 8.0 (Supplementary Figure S6b), following the equation E(V) = −0.064 pH + 0.63 (R^2^ = 0.9961). The obtained slope of −0.64 mV/pH is close to the theoretical Nernstian value (−59.2 mV/pH). According to the Nernst equation, this result confirms that the electrochemical redox process involves the transfer of an equal number of protons and electrons (1:1 ratio).

Finally, the accumulation time significantly influenced the sensor’s response to DA, with peak current increasing from 1 to 3 min before declining at longer durations, likely due to secondary reactions and surface passivation by DA oxidation, and adsorption and polymerization reactions of the by-products.

### 3.3. Dopamine Determination Under Optimized Conditions

**Figure 6:**
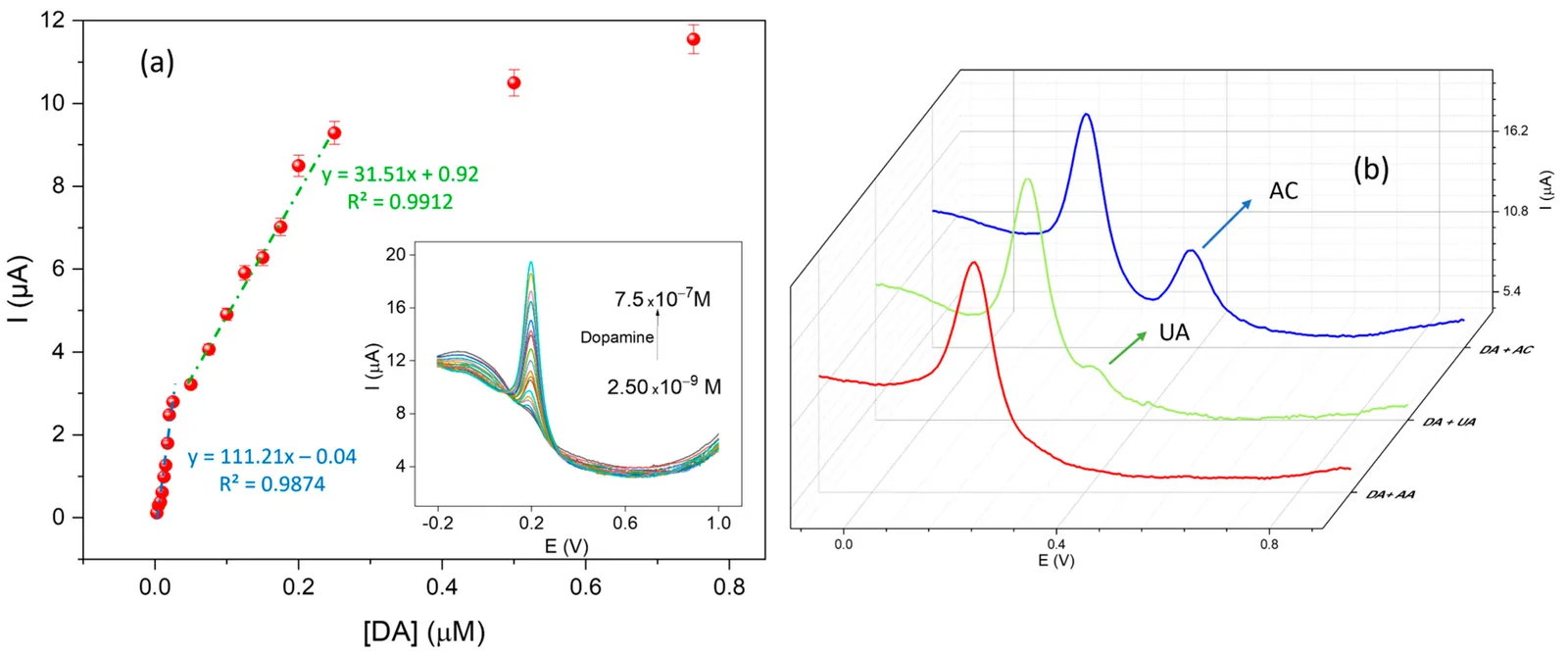
(**a**) Calibration curve and raw differential pulse voltammograms (DPV) (inset) for DA with electrodes prepared with a casting solution of 7 µL, drying time of 2 h, and an accumulation time of 3 min in PBS with a pH of 7.0. (**b**) Raw DPV response of the modified electrode for DA (1.75 × 10^−7^ M) detection in the presence of potential interferents (1 × 10^−3^ M): ascorbic acid (AA), uric acid (UA), and acetaminophen (AC). Red, green, and blue curves represent DA + AA, DA + UA, and DA + AC, respectively.

The GCE/Nafion(rGox/MNPs@PPy) electrodes, under optimized conditions, were used for sensing DA in the range of concentration from 2.5 × 10^−9^–7.5 × 10^−6^ M, exhibiting two linear ranges. Figure 6a illustrates the raw DPV curves and the current response against DA concentration. The sensor presented a positive current correlation with DA concentration in two different ranges, highlighting its versatility and sensitivity. The first range, from 5.4 × 10^−9^ to 2.5 × 10^−8^ M, enabled detection of low DA concentrations, which is crucial for applications in some neurological disorders such as Parkinson’s disease, psychiatric conditions like depression and Attention Deficit Hyperactivity Disorder, metabolic diseases, or as a side effect of certain medications. The second range, extending from 2.5 × 10^−8^ to 2.5 × 10^−7^ M, corresponds to the typical dopamine levels found in biological samples, making the sensor ideal for routine analysis. From 2.5 × 10^−7^ to 7.5 × 10^−6^ M, the sensor could detect elevated dopamine concentrations that may arise in pathological states or experimental conditions; however, in this range, the response does not follow a linear fit. The dilution of samples from patients with pathologies presenting high dopamine concentrations, to bring them within one of the sensor’s linear adjustment ranges, ensures accurate quantification. Thus, the sensor’s outstanding performance is attributed to the combined properties of its components. The rGOx enhances the conducting and charge-transfer properties, increases the surface area, and improves the electrical conductivity of the composite, while MNPs/PPy facilitate electron transfer and further enhance electrochemical activity. On the contrary, the Nafion coating provides selectivity, minimizing interference from other molecules. Together, these features make the GCE/Nafion(rGOx/MNPs@PPy) sensor an effective and reliable tool for DA detection across a wide range of concentrations in diverse applications. The calibration curve for DA exhibits two main distinct linear regimes with a sharp transition around 0.025 µM, followed by a final section with a clear saturation effect (a phenomenon well-documented for electroanalytical systems). As illustrated in Supplementary Figure S7, the experimental data points display a noticeable systematic divergence from the ideal, pure Langmuir isotherm fit (red curve), particularly underestimating the peak currents in the low-concentration range. This discrepancy represents direct electrochemical evidence that a single, homogeneous adsorption model is insufficient to describe the interface dynamics and is fundamentally driven by a shift in the rate-determining step, transitioning from an initial adsorption-controlled process on high-affinity sites to a mass transport mechanism governed by diffusion once monolayer saturation is reached. In the first linear range where the detection is fundamentally governed by an adsorption-controlled process on the high-affinity active sites of the modified electrode surface (up to 0.025 µM), a remarkably high sensitivity of 111.21 µA/µM is achieved. At these ultra-trace levels, the surface coverage (θ) complies with the linear region of the Langmuir adsorption isotherm, where the strong non-covalent interactions or chemical affinity of the modifier effectively pre-concentrate DA at the electrode/electrolyte interface, drastically enhancing the electron transfer rate.

The exceptionally sharp transition into the second linear range signifies the threshold of monolayer saturation of these high-affinity sites (θ → 1). Beyond this critical concentration (0.025 to 0.25 µM), the electrochemical response switches from an adsorption-controlled regime to a diffusion-controlled mass transport mechanism, or alternatively, involves interaction with secondary lower-affinity sites. According to the classic electroanalytical theory (Fick’s laws of diffusion), the peak current remains linearly proportional to the bulk concentration of DA; however, because diffusion lacks the signal-amplifying benefit of strong surface pre-concentration, the sensitivity drops significantly to 31.51 µA/µM. At even higher concentrations (>0.25 µM), the current plateaus due to total surface saturation and potential blocking effect from the deposition of DA oxidation by-products (such as polydopamine-like films), leading to the observed loss of linearity and the asymptotic stabilization of the global fit. The limit of detection (LOD) for DA (S/N = 3) was determined to be as low as 5.4 × 10^−9^ M, being this value lower than many previous sensors reported in the literature, see Table 2, and confirmed the enhanced response of the composite against DA. This result can be attributed to the superior electrochemical activity/conductivity of rGox combined with the polymer-modified MNPs, showcasing excellent sensitivity against DA.

### 3.4. Interference Study

To evaluate the sensor selectivity for DA detection, various electroactive substances were tested, including some biological compounds such as ascorbic acid (AA) and uric acid (UA), as well as a pharmacological compound such as acetaminophen (AC). This comprehensive analysis aimed to assess potential interferences due to the coexistence of DA with other interfering species sharing similar oxidation potentials, which can significantly impact the sensor’s selectivity towards DA. As shown in Figure 6b, the DPV response of DA was evaluated in the presence of potential interferents at pH 7.0 to verify the anti-interference efficiency of the modified electrode. At this physiological pH, electrochemical behavior is strongly governed by electrostatic interactions with negatively charged sulfonate groups of the Nafion-modified composite. AC (pK_a1_ ~ 9.7) remains predominantly neutral and is unaffected by the polymer’s charge, displaying distinct and well-resolved oxidation peaks that ensure no significant overlap with DA. Conversely, AA (pK_a1_ ~ 4.1) and UA (pK_a1_ ~ 5.4) exist entirely as anionic species at a pH of 7.0. Due to the powerful electrostatic repulsion exerted by the Nafion film, the transport to the electrode surface is drastically restricted; consequently, AA yielded no measurable response, while UA exhibited only a negligible, minor peak safely separated from the analyte. These findings underscore the excellent perm-selectivity and anti-interference performance enabled by the modified electrode. In addition, the electrode current towards DA at a concentration of 1.75 × 10^−7^ M in the presence of previous interferences at a concentration of about 1 × 10^−3^ M in PBS (0.1 M, pH = 7.0) was registered. The results presented in Supplementary Figure S8 demonstrate that the GCE/Nafion(rGox/MNPs@PPy) electrode exhibits outstanding selectivity, even in the presence of interfering species at concentrations four orders of magnitude higher than that of DA. These findings highlight the sensor’s remarkable ability to selectively detect DA, underscoring its exceptional performance in complex matrices. Upcoming studies will investigate the influence of biologically relevant metal ions (e.g., Fe^3+^, Cu^2+^, Zn^2+^) on DA detection, considering their potential to interfere through chemical oxidation or complexation mechanisms.

### 3.5. Intra-Electrode Repeatability and Inter-Electrode Fabrication Reproducibility

The intra-electrode repeatability and reusability of the sensor were assessed by measuring its response to a DA concentration of 1 × 10^−5^ M. Supplementary Figure S9a illustrates the normalized and sequential responses of a single sensor tested seven times under identical conditions. Between successive measurements, the sensor was subjected to a cleaning process for 20 min in 2 M PBS. The calculated coefficient of variation for the repeatability tests was 7.9%. In addition, the inter-electrode fabrication reproducibility was evaluated using seven independently assembled sensors following the same preparation protocol. Supplementary Figure S9b displays the results, with a coefficient of variation of 7.4% observed across the different devices. These findings highlight the high reliability and reproducibility of the fabrication process. The results obtained in this study validate the use of this sensor for analytical applications, demonstrating its precision, intra-electrode repeatability, and reliable fabrication under the tested conditions.

### 3.6. Applications to Real Samples Analysis

To further assess the validity of the method and the nanocomposite, real samples were analyzed using the standard addition method. First, 1 mL of each real sample was filtered and then diluted in 99 mL using 0.1 M PBS pH = 7.0. The samples were diluted in a 1:99 ratio with PBS to minimize potential interference from other components in the sample, ensuring optimal sensor performance for DA detection. Next, the diluted samples were spiked with specific amounts of DA and subjected to detection. Recovery rates for spiked samples ranged from 74% to 116%, as shown in Table 3. These experimental results confirm the feasibility and effectiveness of the method in practical applications.

## 4. Conclusions

In this study, we developed a unique sensing platform that integrates electrochemically reduced graphene oxide (rGOx), with polypyrrole-functionalized Fe_3_O_4_ magnetic nanoparticles (MNPs@PPy) and Nafion. This multifunctional composite combines the high conductivity of rGOx, the enhanced electrochemical response and magnetic properties of MNP@PPy, and the selective ion-exchange capability of Nafion to enhance sensor performance. The resulting Nafion(rGox/MNPs@PPy) composite was structurally and electrochemically characterized using XRD, FT-IR, Raman spectroscopy, TG, EIS, SEM, EDS, and TEM analysis. The reduction treatment improved the material’s conductivity and electron transfer, yielding superior electrochemical response. The sensor assembled with this ternary composite showed high sensitivity for dopamine detection, achieving a nanomolar-level limit of detection and effective discrimination against interfering substances such as ascorbic acid, paracetamol, and uric acid. It also demonstrated excellent repeatability and fabrication reproducibility, with coefficients of variation below 8%, along with reliable performance in real biological samples, with recovery rates ranging from 74% to 116%. To our knowledge, this is the first study to report the use of this specific ternary system for DA detection in complex matrices. Its analytical efficiency, precision, and practical applicability make it a promising candidate for future analytical applications. Future work will focus on adapting this sensor platform for the simultaneous detection of multiple neurotransmitters, miniaturization for point-of-care devices, and validation in clinical diagnostic settings.

## Figures and Tables

**Table 1:** Optimized experimental values for conditioning step and for DPV determination of DA.

Pre-Treatment		DPV			
pH	nº Cycle	Casting Solution	Accumulation Time	Drying Time	pH
3.0	6	7 µL	3 min	2 h	7.0

**Table 2:** Comparison with other electrochemical sensors for the determination of dopamine.

Modified Electrode	Method	Linearity (M)	LOD (M)	References
MIPs/MXene/GCE	DPV	0.50–10 × 10^−6^	0.27 × 10^−6^	[66]
S-MoSe_2_/NSG/Au/MIPs/GCE	DPV	0.05–100 × 10^−6^	0.02 × 10^−6^	[67]
PPy-MIP/ZrO_2_@C/NPG/GCE	DPV	0.005–100 × 10^−6^	3.3 × 10^−10^	[68]
GO/PPy/CPE	DPV	0.064–200 × 10^−6^	10 × 10^−9^	[69]
3D-PS-doped CNHN/SPCE	SWV	10–500 × 10^−9^	7.8 × 10^−9^	[70]
CoNi-SPE	bubble-based electrochemical chip/DPV	1 × 10^−6^–10 × 10^−6^ and 10 ×10^−6^–70 × 10^−6^	0.06 × 10^−6^	[71]
GCE/P(*p*-PDA)-MWCNTs/AgNPs	DPV	4.0−800 × 10^−6^	0.048 × 10^−6^	[72]
PEDOT-PPy	DPV	5 × 10^−9^ to 200 × 10^−6^	5 × 10^−9^	[73]
MnS/Co_3_S_4_-ErGO/GCE	SDLSV	6.0 × 10^−9^ to 20.0 × 10^−6^	2.0 × 10^−9^	[74]
OPPy/ERGO/GCE	DPV	4 × 10^−7^–5.2 × 10^−4^	2 × 10^−7^	[41]
Fe_3_O_4_@PPy/rGO/GCE	DPV	up to 1 × 10^−4^	6.3 × 10^−8^	[43]
Tyr/Pg-Ni Fe_2_O_4_/rGO/ITO	CV	1 × 10^−6^–3 × 10^−4^	4.6 × 10^−8^	[75]
GQD/CuCo_2_O_4_/CPE	DPV	5 × 10^7^–1.5 × 10^−4^	4 × 10^−9^	[76]
GCE/Nafion(rGox/MNPs@PPy)	DPV	5.4 × 10^−9^–2.5 × 10^−8^ 2.5 × 10^−8^–2.5 × 10^−7^ 2.5 × 10^−7^–7.5 × 10^−7^	5.4 × 10^−9^	Current work

MIPs: molecularly imprinted polymer; MXene: two-dimensional (2D) transition metal carbides, nitrides, and carbonitrides, derived from layered MAX phases; GCE: glassy carbon electrode; S-MOSe_2_: Sulfur-doped Molybdenum diselenide; NSG: nitrogen-doped graphene; PPy: polypyrrole; GO: graphene oxide; 3D-PS-doped CNHN: three-dimensional phosphorous and sulfur resembling g-C_3_N_4_ hornet’s nest; P(*p*-PDA: poly(*p*-phenylenediamine); MWCNT: multi-walled carbon nanotubes; AgNPs: silver nanoparticles; PEDOT: poly(3,4-ethylenedioxythiophene); MnS/Co_3_S_4_: hollow tubular manganese-cobalt sulfide; ErGO: electrochemically reduced graphene oxide; OPPy: Overoxidized Polypyrrole; Fe_3_O_4_@PPy/rGO: Magnetite@Polypyrrole/reduced graphene oxide; Tyr/Pg-NiFe_2_O_4_/rGO/ITO: parallelogram NiFe_2_O_4_-modified reduced graphene oxide nanocomposite on Indium Tin Oxide electrode; GQD/CuCo_2_O_4_/CPE: CuCo_2_O_4_/graphene quantum dots-modified carbon paste electrode; DPV: differential pulse voltammetry; SWV: Single Wave Voltammetry; CV: Cyclic Voltammetry; SDLSV: Second-order Derivative Linear Sweep Voltammetry.

**Table 3:** Determination of DA in human urine and blood samples.

Sample	Added (M)	Found (M)	Recovery (%)	RSD * (%) (n = 3)
Blood	_ 7.5 × 10^−9^ 1.0 × 10^−8^ 1.25 × 10^−8^	_ 5.62 × 10^−9^ 1.03 × 10^−7^ 1.45 × 10^−8^	_ 74.93 103 116	_ 3.62 1.31 2.3
Urine	_ 1.0 × 10^−8^ 1.5 × 10^−7^	_ 8.3 × 10^−9^ 1.49 × 10^−7^	_ 83 99.33	_ 2.22 0.85

* RSD = relative standard deviation.

## Data Availability

The data presented in this study are available on request from the corresponding author.

## Supplementary Materials

The following supporting information can be downloaded at: https://www.mdpi.com/article/10.3390/mi17080908/s1. Table S1. EDS elemental quantification data of the Nafion(Gox/MNPs@PPy) composite film. Figure S1. Influence of the number of cycles in 0.1 M PBS (pH 5) during the electrochemical reduction step in the response of the electrode in 0.1 M PBS (pH 7) containing 10^−5^ M DA (casting solution: 5 µL; drying time: 1h; accumulation time: 3 min; pH during DPV: 7). Figure S2. Influence of the pH in 0.1 M PBS during the electrochemical reduction step using CV (n = 6) in the response of the electrode in 0.1 M PBS (pH 7) containing 10^−5^ M DA (casting solution: 5 µL; drying time: 1h; accumulation time: 3 min; pH during DPV: 7). Figure S3. Influence of the amount of matrix deposited on the electrode in PBS (pH 7, 0.1M) contained 10^−5^ M of DA. Figure S4. Influence of the accumulation time in the response of the electrode in PBS (pH 7, 0.1M) contained 10^−5^ M of DA. Figure S5. Influence of drying time in the response of the electrode in PBS (pH 7, 0.1M) contained 10^−5^ M of DA. Figure S6. Influence of the pH in the response of the electrode in PBS (0.1M) contained 10^−5^ M of DA. (a) Raw DPV data and (b) Linear relationship regression between applied potential and pH. Figure S7. Global non-linear Langmuir isotherm fit (red curve, R^2^ = 0.984) displaying systematic divergence due to mechanistic shifts, and an asymptotic plateau above 0.25 µM (dashed line) from surface blocking. Inset plot: Close-up of the dual-linear ranges showcasing the sharp transition at 0.025 µM; the blue line marks the adsorption-controlled regime up to full monolayer coverage (θ→1), purple arrow), while the green line tracks the subsequent diffusion-controlled mass transport mechanism. Figure S8. Normalized DPV peak currents (%) of DA (1.75 × 10^−7^ M) in the presence of AA, AC, and UA as potential interferents (1 × 10^−3^ M) in PBS (pH 7.0). The current response of DA alone is set as 100%. Error bars represent the standard deviation (n = 3). Figure S9. Intra-electrode repeatability (a) and inter-electrode fabrication reproducibility (b) of the GCE/Nafion(rGox/MNPs@PPy) electrode toward a concentration of 1 × 10^−5^ M DA.
