# Supplementary material for: Electrochemical Sensing of Dopamine with a Nafion-Coated Reduced Graphene Oxide/Polypyrrole-Functionalized Magnetic Nanoparticles Composite
Source: Micromachines (Basel). 2026 Jul 29;17(8):908. doi: 10.3390/mi17080908 (PMC13515962; doi:10.3390/mi17080908)
Supplement: Supplementary file 1 [file micromachines-17-00908-s001.zip › micromachines-4419543-supplementary.pdf]

Electrochemical Sensing of Dopamine with a Nafion-Coated Reduced Graphene Oxide/Polypyrrole-Functionalized Magnetic Nanoparticles Composite

Afef Dhaffouli [1,2], Paul E.D. Soto-Rodríguez [3], Soledad Carinelli [4], Houcine Barhoumi [1], José Luis González-Mora [4], Pedro A. Salazar-Carballo [4,*]

[1] Laboratory of Interfaces and Advanced Materials, Faculty of Sciences of Monastir, University of Monastir, Monastir 5000, Tunisia
[2] Department of Chemistry, Faculty of Sciences of Gafsa, University of Gafsa, Gafsa 2100, Tunisia
[3] Department of Physics, Instituto de Estudios Avanzados (IUDEA), University of la Laguna, 38002 La Laguna, Spain
[4] Laboratory of Sensors, Biosensors and Advanced Materials, Faculty of Health Sciences, University of La Laguna, Campus de Ofra s/n, 38071 La Laguna, Spain
* Correspondence: psalazar@ull.edu.es

Table S1. EDS elemental quantification data of the Nafion(Gox/MNPs@PPy) composite film.

Element	Line Type	Wt%	Wt% Sigma	Atomic %
C	K series	56.67	0.11	69.06
N	K series	2.62	0.12	2.74
O	K series	26.52	0.08	24.26
Al	K series	0.10	0.01	0.05
Si	K series	0.06	0.01	0.03
P	K series	0.07	0.01	0.03
S	K series	0.90	0.01	0.41
Cl	K series	0.24	0.01	0.10
K	K series	0.02	0.01	0.01
Fe	K series	12.42	0.04	3.26
Cu	K series	0.14	0.02	0.03
Pb	M series	0.23	0.04	0.02

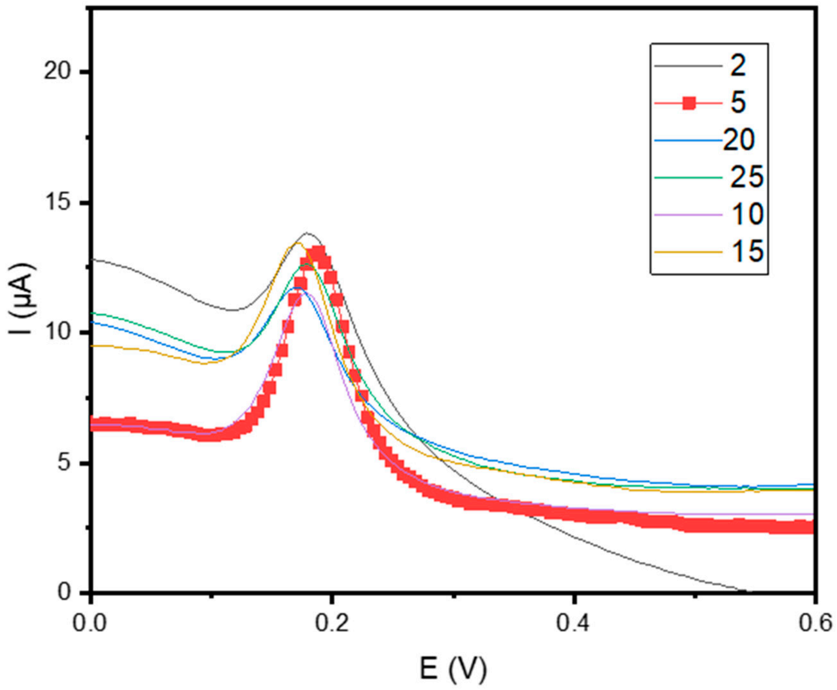

Figure S1. Influence of the number of cycles in 0.1 M PBS (pH 5) during the electrochemical reduction step in the response of the electrode in 0.1 M PBS (pH 7) containing 10^{-5} M DA (casting solution: 5 µL; drying time: 1h; accumulation time: 3 min; pH during DPV: 7).

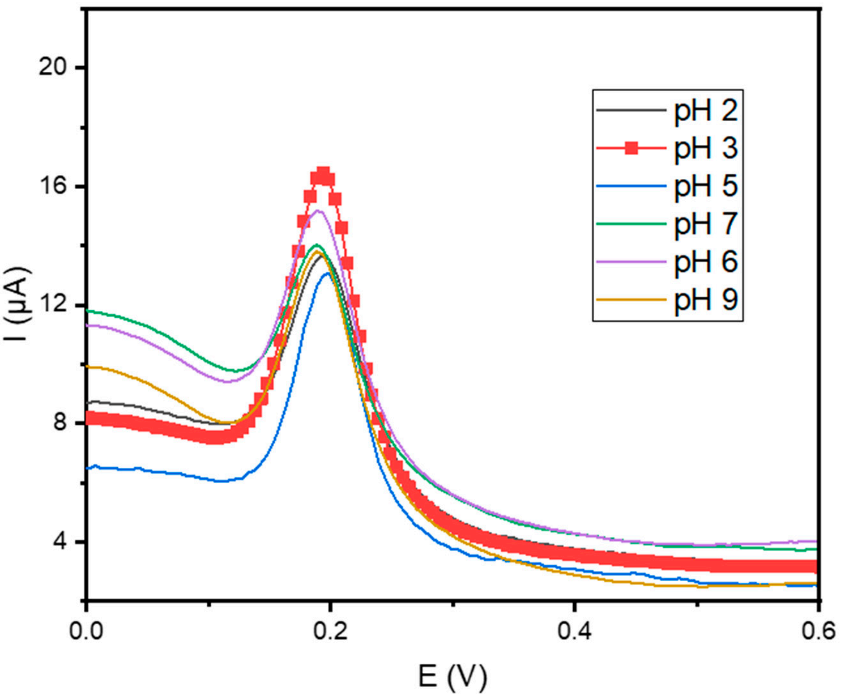

Figure S2. Influence of the pH in 0.1 M PBS during the electrochemical reduction step using CV (n = 6) in the response of the electrode in 0.1 M PBS (pH 7) containing 10^{-5} M DA (casting solution: 5 µL; drying time: 1h; accumulation time: 3 min; pH during DPV: 7).

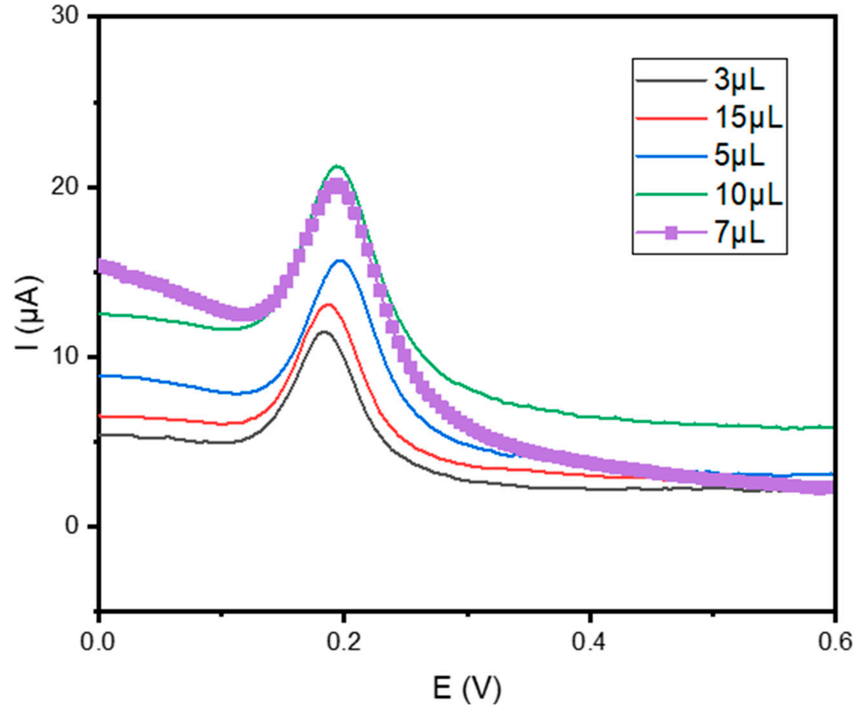

Figure S3. Influence of the amount of matrix deposited on the electrode in PBS (pH 7, 0.1M) contained 10^{-5} M of DA.

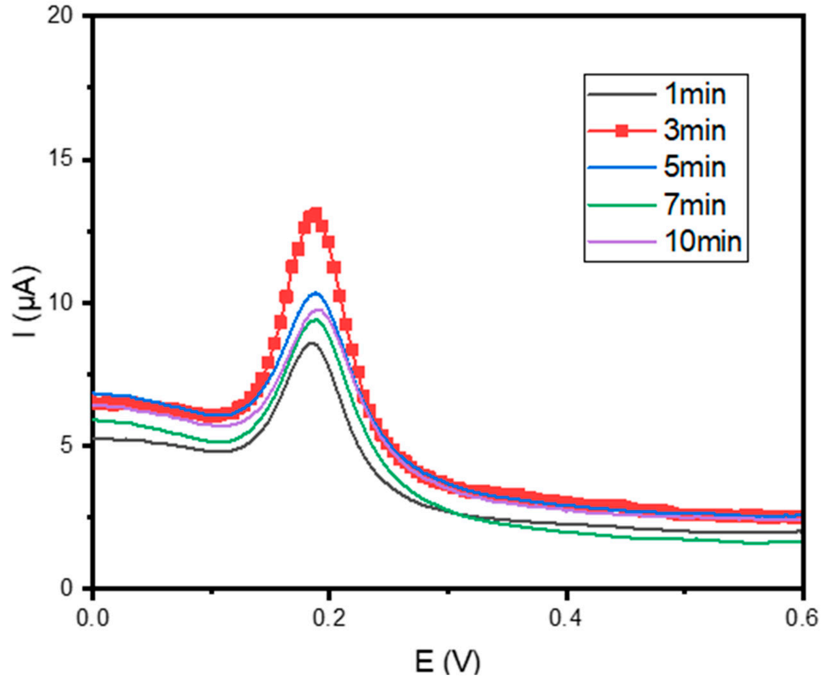

Figure S4. Influence of the accumulation time in the response of the electrode in PBS (pH 7, 0.1M) contained 10^{-5} M of DA.

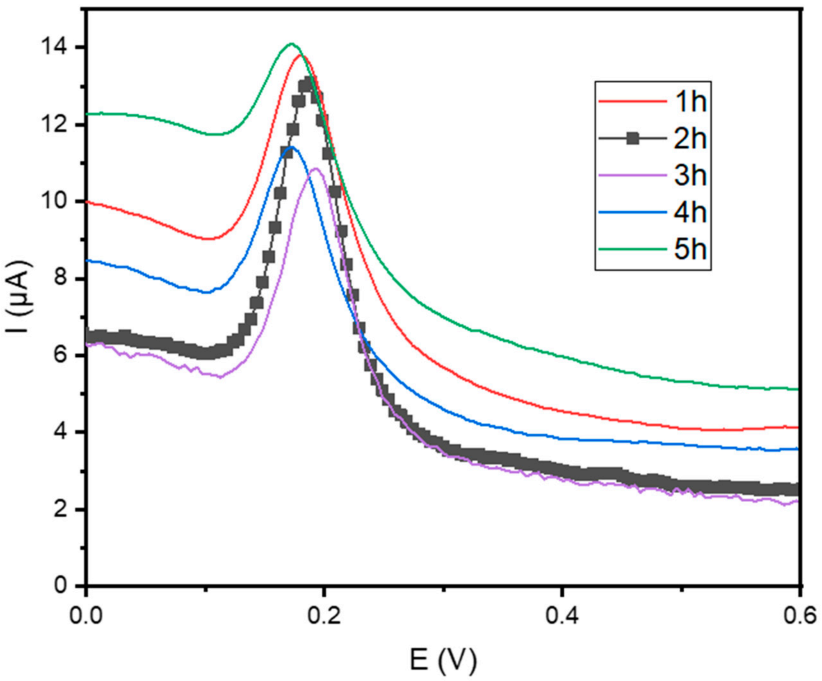

Figure S5. Influence of drying time in the response of the electrode in PBS (pH 7, 0.1M) contained 10^{-5} M of DA.

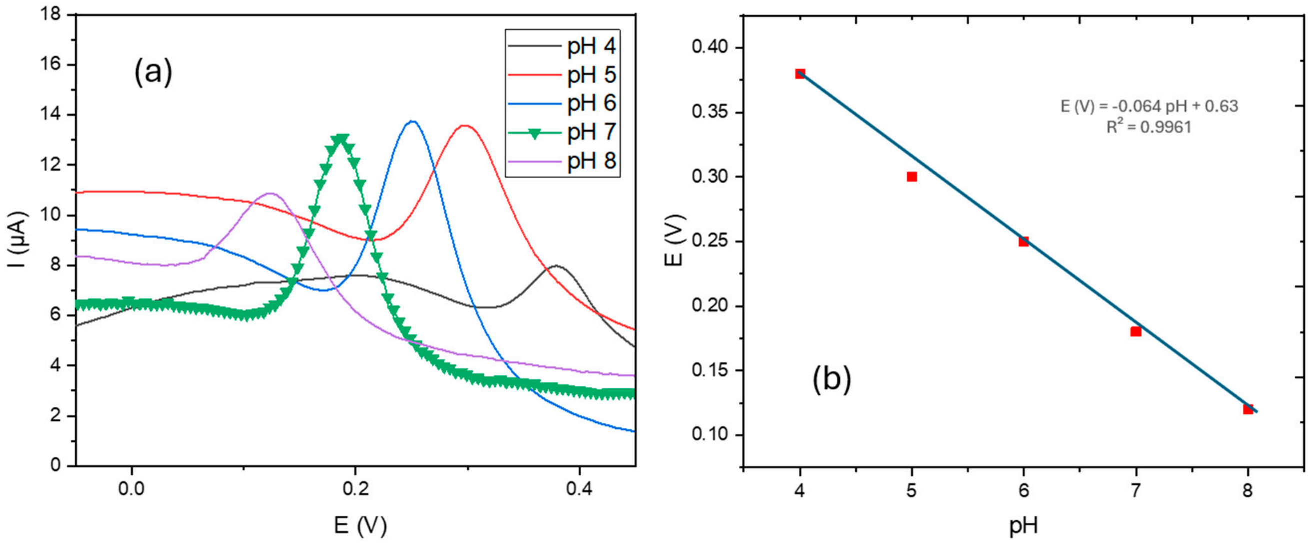

Figure S6. Influence of the pH in the response of the electrode in PBS (0.1M) contained 10^{-5} M of DA. (a) Raw DPV data and (b) Linear relationship regression between applied potential and pH.

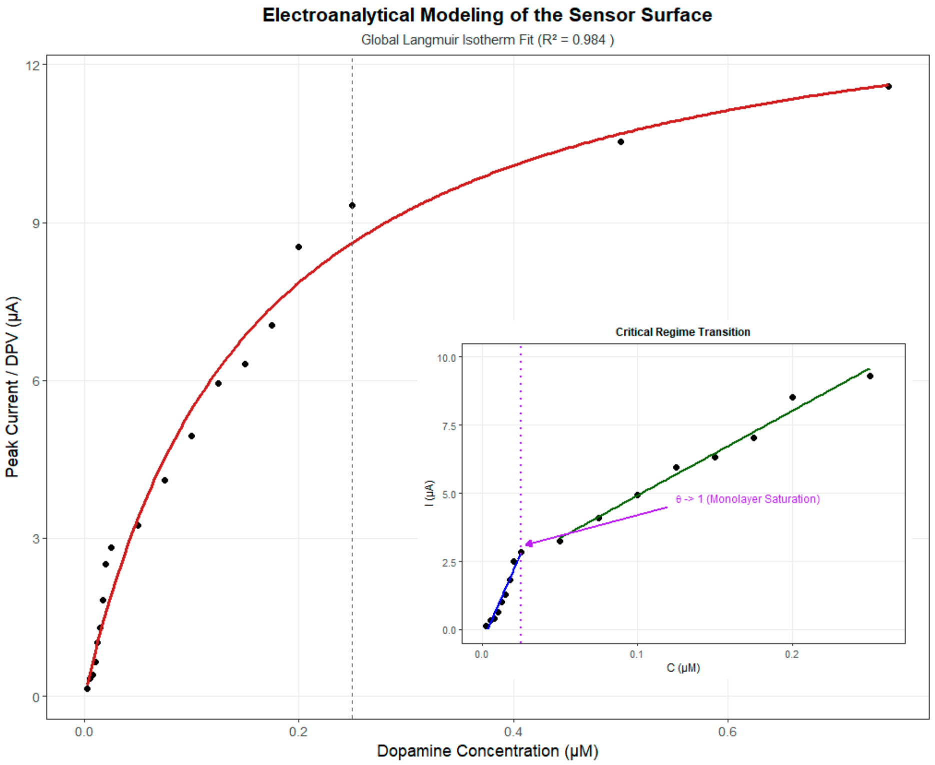

Figure S7. Global non-linear Langmuir isotherm fit (red curve, $R^2 = 0.984$) displaying systematic divergence due to mechanistic shifts, and an asymptotic plateau above 0.25 µM (dashed line) from surface blocking. **Inset plot:** Close-up of the dual-linear ranges showcasing the sharp transition at 0.025 µM; the blue line marks the adsorption-controlled regime up to full monolayer coverage ($\theta \rightarrow 1$), purple arrow), while the green line tracks the subsequent diffusion-controlled mass transport mechanism.

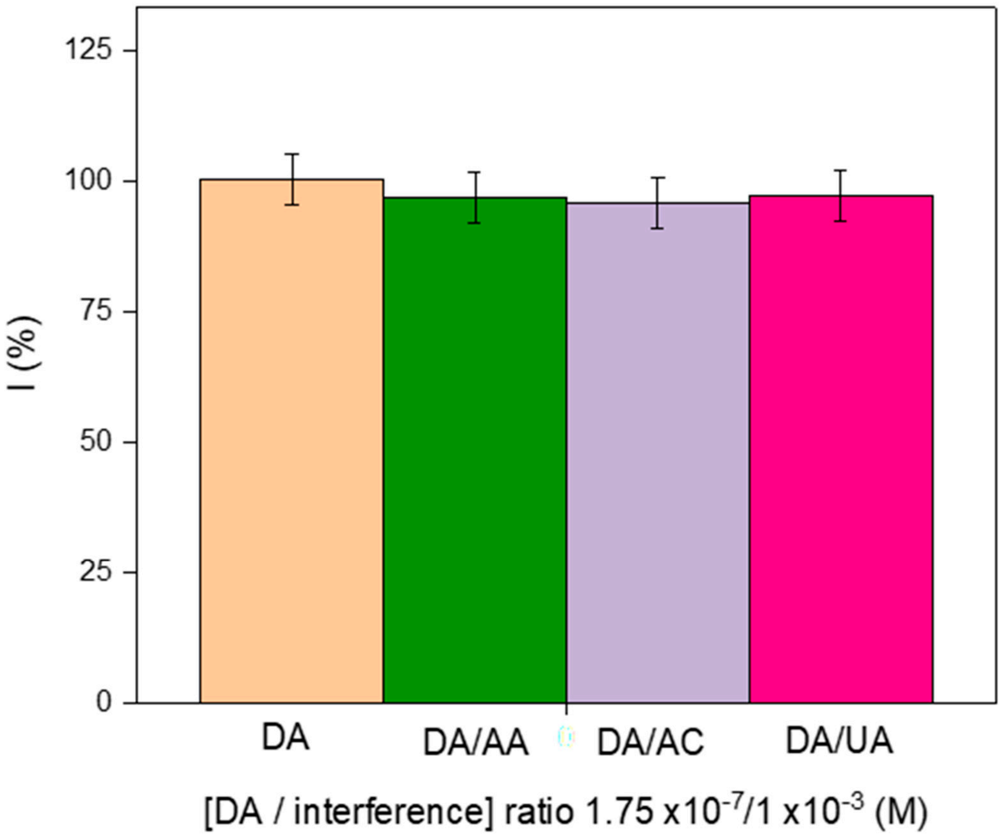

Figure S8. Normalized DPV peak currents (%) of DA (1.75×10^{-7} M) in the presence of AA, AC, and UA as potential interferents (1×10^{-3} M) in PBS (pH 7.0). The current response of DA alone is set as 100%. Error bars represent the standard deviation (n = 3).

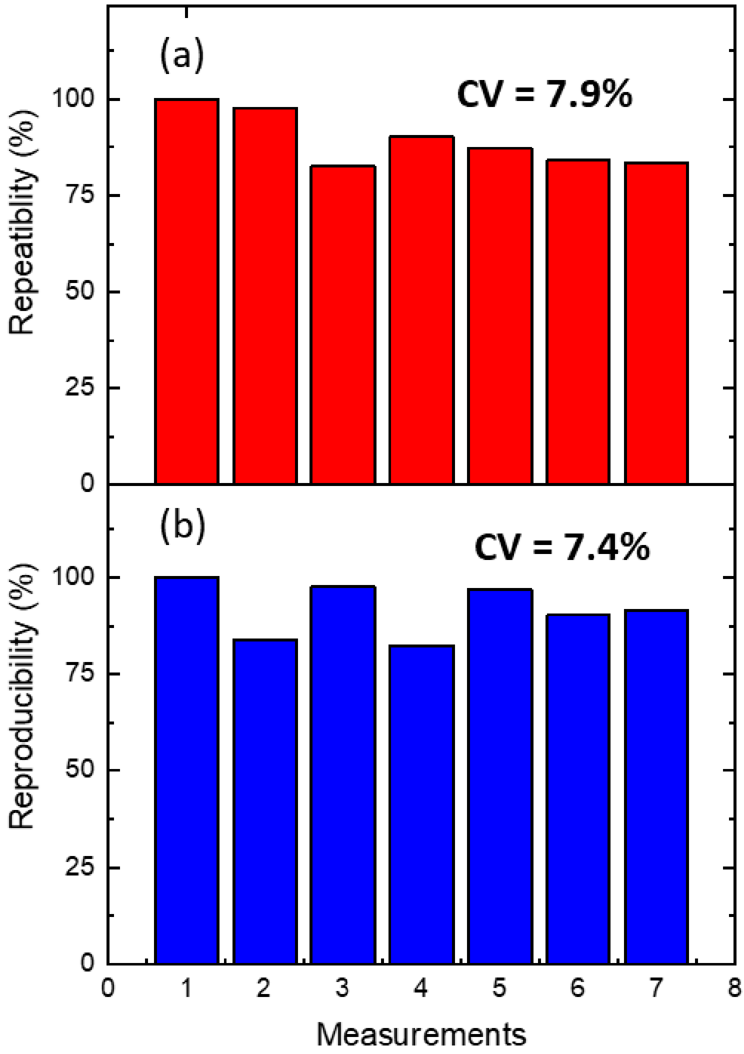

Figure S9. Intra-electrode repeatability (a) and inter-electrode fabrication reproducibility (b) of the GCE/Nafion(rGox/MNPs@PPy) electrode toward a concentration of 1×10^{-5} M DA.
